# Multi-scale fusion SDAM-net empowered by physics-informed conditional spectral augmentation enabling spectral feature assignment for the concurrent detection of nitrogen and sulfur in ship fuel oil

**DOI:** 10.1039/d6ra04810b

**Published:** 2026-08-28

**Authors:** Haoze Tian, Yanan Zhang, Di Wu, Chuqiao Hu, Peilun Qiu, Jianqiao Liu, Ce Fu

**Affiliations:** a College of Marine Electrical Engineering, Dalian Maritime University Dalian 116026 Liaoning PR China; b College of Information Science and Technology, Dalian Maritime University Dalian 116026 Liaoning PR China jqliu@dlmu.edu.cn fu_ce@dlmu.edu.cn

## Abstract

The quantitative detection of nitrogen (N) and sulfur (S), the primary pollutant elements in marine fuel oil, is essential for global atmospheric environmental protection. However, the efficient *in situ* detection of these elements is hindered by the reliance of current methods on shore-based technical support and the severe spectral entanglement within complex fuel matrices. Herein, we demonstrate a multi-scale feature-fusion multi-task network (SDAM-Net) that decouples the fluorescence spectra of tin dioxide quantum dots (SnO_2_ QDs) for the efficient *in situ* detection of N and S in ship fuel oil. A physics-informed conditional Wasserstein generative adversarial network (CWGAN) is proposed to reconstruct a continuous spectral manifold to overcome the limitations of insufficient training samples. This conditional generative strategy mitigates the combinatorial sparsity of N and S concentrations in the experimental dataset, thereby resolving predictive instability caused by sample-scarce regions. The incorporation of an interpretability module based on SHapley Additive exPlanations (SHAP) assigns the extracted salient features to specific microscopic spectral responses. This feature assignment confirms the successful separation of entangled spectra and elimination of chemical crosstalk, validating the model decoupling strategy. Performance evaluation on the test dataset confirms that the SDAM-Net-based framework achieves high detection accuracy for N and S through spectral decoupling, with root mean square errors (RMSEs) reaching 0.0454% and 0.0376%, respectively. This method establishes a robust paradigm for the efficient *in situ* detection of N and S in marine fuel oil, providing a crucial detection platform for atmospheric environmental protection.

## Abstract 2

The SDAM-Net framework enables efficient *in situ* detection of N and S in marine fuel oils *via* spectral decoupling. It delivers high quantitative accuracy with RMSEs of 0.0454% and 0.0376% for N and S, respectively.

## Graphical abstract

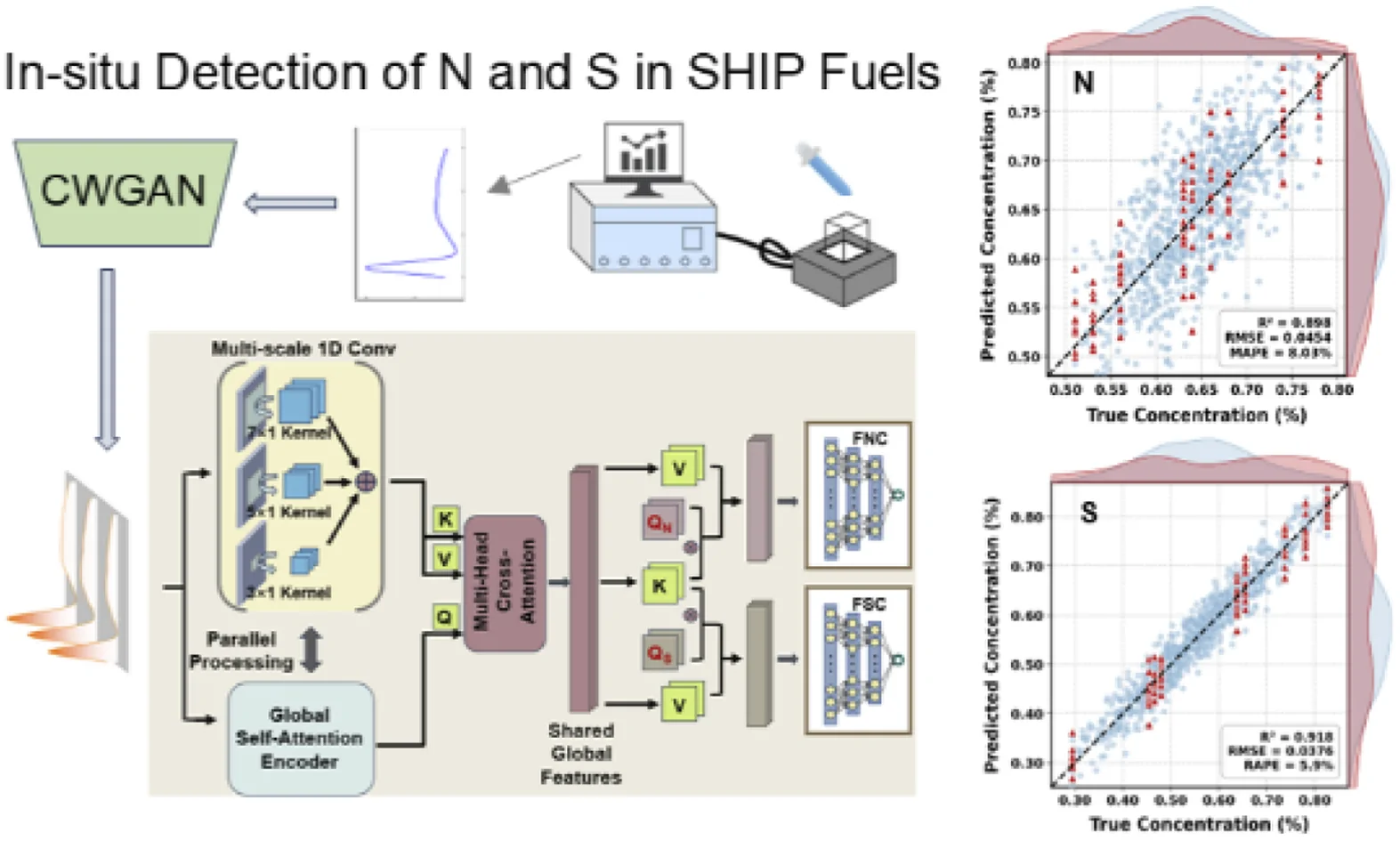

## 1. Introduction

The quantitative detection of nitrogen (N) and sulfur (S) in marine fuel oil plays a crucial role in global atmospheric environmental protection. The excessive emission of nitrogen oxides (NO_*x*_) and sulfur oxides (SO_*x*_), the primary pollutants in ship exhaust emissions, exacerbates acid rain and the greenhouse effect and poses a severe threat to human respiratory health and marine ecological balance.^1,2^ The International Maritime Organization (IMO) has imposed stringent limits on the concentrations of these two primary harmful elements in marine fuel oil, necessitating the establishment of more rigorous emission monitoring systems on a global scale.^3,4^ However, current detection technology development encounters extended detection cycles. Traditional quantitative elemental methodologies rely on shore-based laboratory technical support.^5^ This methodology necessitated cumbersome sampling and delivery procedures, and these procedural impediments prolonged vessel turnaround cycles and escalated the associated operational costs. The implementation of efficient *in situ* detection eliminated shore-based reliance, offered immediate and accurate fuel compliance feedback for ocean-going vessels, and yielded a substantial increase in the detection efficiency of regulatory departments.^6,7^

The introduction of a tin dioxide quantum dot (SnO_2_ QD) fluorescence probe system provides an efficient spectroscopic strategy for the *in situ* quantitative detection,^8,9^ potentially for N and S in marine fuel oil. Furthermore, metal doping modifications optimized the pristine nanostructures to enhance their overall performance,^10–17^ especially their element selective responses.^18^ For example, Ni–SnO_2_ QDs were utilized for the independent quantitative detection of S,^19^ while Nb–SnO_2_ QDs were employed for the specific detection of N content.^20^ In recent years, AI-driven methodologies, including machine learning and deep learning, have provided innovative strategies^21,22^ not only for material discovery^23–26^ and microstructure design^27–30^ but also for performance optimization^31,32^ and mechanistic interpretation.^33,34^ The integration of a deep learning network framework for the recognition of S spectral features within the tin oxide quantum dot system provides a viable solution for *in situ* detection.^35–37^ However, the mutual coupling of the N and S response signals in the spectrum generated severe signal interference and obscured the precise quantification of individual elemental concentrations.^38^ This spectral entanglement^39–41^ precludes existing technologies from executing synchronous dual-element detection and compromises *in situ* detection efficiency.^42^ The effective decoupling of coupled fluorescence signals^43–46^ has become an urgent necessity for the realization of efficient *in situ* N and S detection.^47^

Multi-scale feature extraction achieves the adaptive extraction of local and global spectral information across diverse frequency bands and has found extensive application in the domain of spectral detection for complex matrices and coupled signal processing.^48–51^ For example, a multi-scale convolutional neural network with multi-head attention mechanisms and a multi-task processing head achieved the simultaneous detection of multiple quality parameters in rapeseed oil through ultraviolet fluorescence spectroscopy.^52^ This network structure achieved the decoupling of coupled signal components within the spectral information and demonstrated superior joint detection performance during the simultaneous prediction of multiple quality indicators for rapeseed oil. Network models based on multi-scale feature fusion have achieved the deep aggregation of multi-scale and multi-level spectral feature information within the near-infrared spectroscopy (NIR) detection domain. This multi-scale feature-fusion architecture achieved the precise differentiation of coupled spectral signals for complex wood species and enhanced the classification prediction accuracy.^53^ Additionally, methods adopting a symmetric multi-scale feature-fusion architecture for hyperspectral images and Raman spectra classification successfully resolved weak feature extraction bottlenecks under high-noise backgrounds and demonstrated the generalization advantages of this technology in processing of complex spectral coupling.^54^ The introduction of a multi-scale feature extraction network with a multi-task processing head holds the potential to resolve the coupling problem of N and S spectral signals during *in situ* detection.^55–58^

Herein, we demonstrate a multi-scale feature-fusion multi-task network (SDAM-Net) to separate coupled N and S response signals within the fluorescence spectra from SnO_2_ QDs. The multi-task framework SDAM-Net combines parallel multi-scale convolutional neural networks (MCNNs) and parallel global attention (P-GA) mechanisms with cross-attention modules for the extraction of spectral features across diverse scales. Multi-scale fused feature information is transmitted to decoupled cross-attention (DCA) heads to facilitate the dynamic adjustment of attention resources according to the feature requirements of N and S detection tasks. The integration of real physical constraints into a conditional Wasserstein generative adversarial network (CWGAN) ensures the alignment of spectral generation with physical laws and addresses the combinatorial sparsity of N and S concentrations through a conditional generative strategy. An interpretability module based on SHapley Additive exPlanations (SHAP) assigns salient features within N and S prediction tasks to respective real spectral response curves and restores the network decoupling process through the explanation of fluorescence response properties across diverse spectral bands. The model was evaluated for the quantitative detection of N and S on the constructed test dataset. This method holds the potential to overcome the inability to decouple N and S signals within the spectrum, achieving efficient *in situ* detection of N and S in marine fuel oil.

## 2. Methods

### 2.1 Synthesis of SnO_2_ QDs

SnO_2_ QDs were prepared through a one-step hydrothermal method. The synthesis was initiated with the dissolution of 0.077 g of thiourea (CH_4_N_2_S) in 50 mL of deionized water under continuous stirring to achieve full dissolution. Subsequently, 2.257 g of stannous chloride dihydrate (SnCl_2_·H_2_O) was added, followed by a 24-hours agitation phase with a magnetic stirrer at room temperature. The resulting SnO_2_ precursor solution was transferred to a Teflon-lined stainless steel autoclave. The hydrothermal reaction was carried out at 180 °C for 6 hours. The autoclave system was cooled to room temperature upon completion. The final SnO_2_ QDs was purified by multiple washes with absolute ethanol.

### 2.2 Fluorescence detection

The procedure for raw data collection and detection involved specific preparation phases. Dilution of 10 µL of the SnO_2_ quantum dot solution with 10 mL of absolute ethanol produced the diluted tin dioxide quantum dot ethanol solution. The dissolution of 0.05 g of the fuel oil sample required 5 mL of *n*-hexane. The subsequent addition of 5 mL of absolute ethanol to the fuel oil sample constituted the initial dilution step. A further dilution of 1.5 mL of the fuel solution at a 1 : 10 volume ratio with ethanol yielded the final diluted fuel solution. The mixture of 60 µL of fuel dilution solutions with varying S contents and 1.8 mL of the diluted SnO_2_ quantum dot solution served as the test samples. A fluorescence spectrophotomete*r* (F96pro, Shanghai Lengguang Technology Co., Ltd, China) was used to record the fluorescence emission spectral data. Ten distinct authentic marine fuel oil formed the evaluation set. The N mass fractions were 0.51%, 0.53%, 0.56%, 0.63%, 0.63%, 0.64%, 0.66%, 0.68%, 0.74%, and 0.78% m m^−1^. The S mass fractions were 0.294%, 0.454%, 0.465%, 0.479%, 0.637%, 0.655%, 0.737%, 0.780%, and 0.826% m m^−1^. Fluorescence spectra for each fuel oil sample were collected over a wavelength range of 251–400 nm at a 1 nm resolution, with 60 replicates acquired per oil type. Prior to model training, the 5 spectra with the highest and the 5 with the lowest maximum emission peak intensities within each sample group were discarded. This step excluded 10 samples per group, yielding a final dataset of 50 spectral records for each fuel oil and an overall total of 500 records for subsequent network training.

### 2.3 Data augmentation

The CWGAN architecture defined the condition vector as a dual-element concentration combination *c =* [*y*_N_,*y*_S_]^*T*^.^59^ The generator aimed to learn the nonlinear mapping from the latent noise space to the real spectral space. The generator received a random noise vector *z ∼* (0,1) from a Gaussian distribution and the concatenated condition label c, and the final output produced a synthetic fluorescence spectral sequence *x̂* = *G*(*z|c*), with a length of 150.

A series of 1D convolutional neural network (CNN) layers and LeakyReLU activation functions constituted the discriminator. Traditional GAN architectures generated a probability value between 0 and 1. The discriminator of the CWGAN removed the sigmoid activation function in the final layer to output a scalar score for the Wasserstein distance approximation.^60^ The discriminator received the real spectrum and the corresponding concentration label c to evaluate the authenticity of the spectral morphology under specific S and N response conditions. A gradient penalty term is introduced in the optimization process to ensure the Lipschitz continuity condition of the network.

The loss function of the discriminator (*L*_D_) is defined as follows:1.1
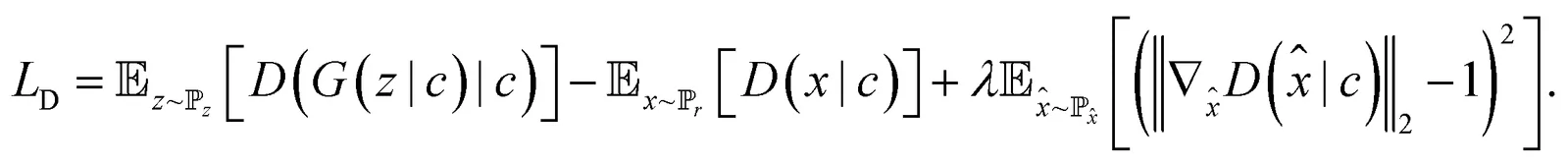
herein, 
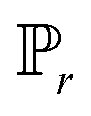
 represents the real spectral data distribution, and 
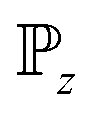
 represents the prior noise distribution. The variable *c* = [*y*_N_,*y*_S_]^*T*^ serves as the conditional vector, indicating the specific concentration combinations of N and S. The symbol *x̂* indicates the interpolated sample, established as a random linear interpolation between the authentic and the generated data points to facilitate the gradient penalty computation. This interpolation is formulated as follows:

1.2 *x̂* = *εx* + (1 − *ε*)*G*(*z*|*c*),

where *ε* ∼ *U*[0,1] is a random scalar drawn from a uniform distribution across the interval [0,1]. Furthermore, the expression ∇*x̂D*(*x̂*|*c*) characterized the gradient for the output of the discriminator with respect to the interpolated sample *x̂* under the specific condition *c*. This rigorous formulation enforced the 1-Lipschitz continuity constraint, guaranteeing the structural stability of the adversarial training process. The scalar *λ* functioned as the gradient penalty coefficient.

The adversarial loss function of the generator (*L*_G_) is simplified to the minimization of the discriminator score for the synthetic samples:1.3
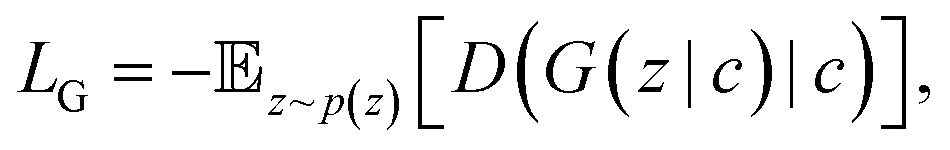
where *z* represents the random noise vector sampled from the prior distribution 
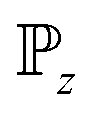
, and *c* denotes the conditional concentration vector. The term *G*(*z*|*c*) symbolizes the synthetic fluorescence spectral sequence. The expression *D*(*G*(*z*|*c*)|*c*) evaluated the authenticity score assigned to the synthetic data by the discriminator.

The incorporation of physical constraints into the CWGAN training objective ensured that the synthetic spectra conformed to the underlying photophysical principles. Within the SnO_2_ QD fluorescence probe system, the primary emission peak intensity (*I*_peak_) exhibited a deterministic dependency on the analyte concentrations: an increase in the N concentration induced an elevation in the spectral peak intensity, whereas an increase in the S concentration triggered a reduction in the peak intensity. The formulation of a physics-informed regularization term (*L*_physics_), based on the partial derivatives of the generated peak intensity with respect to the input conditional concentration vector, enforces these domain-specific laws within the gradient-based optimization:1.4
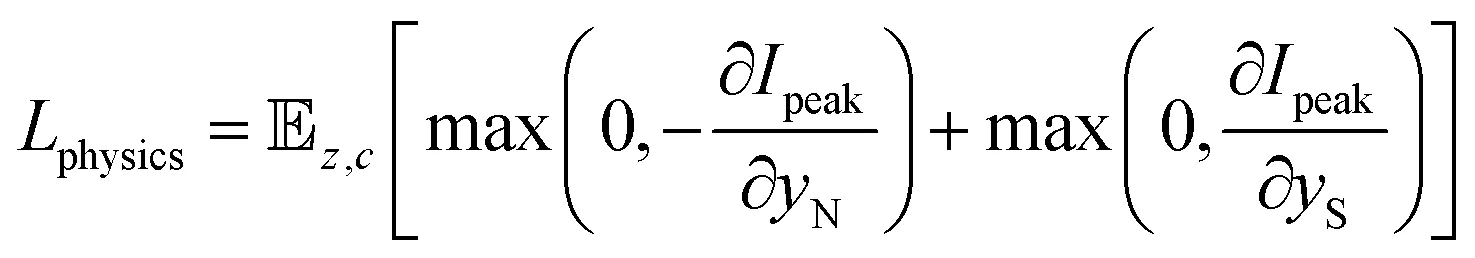
where *y*_N_ and *y*_S_ denote the conditional concentrations of N and S, respectively. This physical loss term applies a directional numerical penalty whenever the generator violates the specified concentration-peak relationships.

As a result, the total loss function of the generator is as follows:

1.5 *L*_G,total_ = *L*_G_ + *λ*_phys_*L*_physics_

here, *L*_G_ represents the standard adversarial loss, and *λ*_phys_ represents the regularization coefficient that scales the physical constraint. This integration guided the generator search space and forced the network to prune unphysical spectral configurations during adversarial training iterations. A constant penalty on non-physical gradient directions enabled the model to reconstruct a robust and physics-informed spectral manifold, even within sample-scarce regions.

The model used the Adam optimizer for alternating adversarial training during the training process. The network generated 10 000 spectral data samples with different N and S combinations within the original concentration boundaries after convergence. The concentration boundaries included a N range of 0.51% to 0.78% m m^−1^ and S range of 0.294% to 0.826% m m^−1^.

### 2.4 Backbone network

The normalized fluorescence spectral sequences within the 251 to 400 nm wavelength range constituted the direct input features for the network, and the overall framework adopted an end-to-end deep learning approach. The normalized feature tensor was fed into the parallel one-dimensional MCNN and the global self-attention encoder. The one-dimensional MCNN employed three parallel CNN columns. An alternating structure of convolution and pooling constituted all three network columns. The three parallel convolutional branches utilized kernel sizes of 7 × 1, 5 × 1, and 3 × 1. A 1 × 2 max pooling layer followed each convolution kernel. The activation module adopted the Gaussian error linear unit (GELU). The fusion of the cascaded feature maps produced a 96-dimensional channel output. This output matched the downstream attention module. The linear mapping module in the global self-attention encoder transformed the original sequence into a 64-dimensional hidden space. The multi-head self-attention core activated 4 attention heads. The residual structure and the layer normalization module ensured forward propagation stability. The multi-head cross-attention (MCA) aggregated the dual-branch feature data. The global encoding output served as the query matrix. The MCNN output constructed the key matrix and the value matrix. The attention head configuration was maintained at 4 groups. The calculation of attention weights adopted the softmax function to map the scaled dot-product results into a probability distribution.^61^ The DCA mechanism received the high-dimensional fused features. The network architecture included a N feature decoupling layer and a S feature decoupling layer. The attention layer generated an element-specific weight matrix. The parameter dimension was maintained at the 64-dimensional standard. The decoupled feature subspace flowed into the dual-branch regression layer. The global average pooling layer compressed the sequence feature data. The pooled output was fed into the multi-layer perceptron (MLP). The initial linear layer executed feature compression from 64 dimensions to 32 dimensions. The activation module used the GELU function^62^ to maintain a consistent non-linear network expression. A dropout strategy with a 20% random drop rate mitigated model overfitting.^63^ The terminal linear layer mapped the features to a one-dimensional real number. The dual-branch network generated the predicted concentration values of N and S. The model training phase adopted the mean squared error (MSE) as the core loss function. The multi-task regression architecture produced two independent error components. The sum of the N concentration prediction error and the S concentration prediction error constituted the total loss value. Parameter optimization was performed using the AdamW optimizer. The weight decay coefficient was 0.01. This configuration introduced an L2 regularization constraint. The backpropagation algorithm executes network weight updates based on the total loss gradient. This weight update minimized the numerical difference between the predicted concentrations and true concentrations.

### 2.5 Training strategy

The original real data comprised 10 fuel combination labels. Each label contained 50 spectral data items. The total count reached 500 one-dimensional spectral data records. Prior to model training, the raw fluorescence spectral data were preprocessed using the min–max scaling method to standardize the fluorescence intensity across all spectral wavelength points into the interval of [−1, 1], thereby achieving the unification of data dimensions. The high acquisition cost and small volume of real spectral samples dictated the absence of an independent validation set in this study. A ten-fold cross-validation strategy ensured the robustness of the model evaluation. The overall dataset division utilized a stratified sampling approach on the entire sample set with real N and S concentration labels. The division process split the samples into ten mutually exclusive subsets of equal size. A mixed test set construction mechanism provided a rigorous evaluation of the quantitative detection performance of the model in the continuous concentration domain. The CWGAN model generated a small batch of high-fidelity synthetic spectral data as a concentration supplement set. This generation targeted the missing N and S concentration combination intervals in the real samples. This supplement set was strictly excluded from feature extraction training in all folds. The subsequent injection of these data into the test set expanded the regression sample volume. The ten-fold cross-validation involved ten independent iterative cycles. Each iteration selected one real sample subset. The procedure combined this selected subset with the untrained synthetic concentration supplement set to construct the mixed test dataset for the current fold. This mixed dataset was used to assess the model performance for the quantitative detection of N and S. The remaining nine real sample subsets served as the benchmark training set.

### 2.6 Ablation experiments

Ablation experiments were performed to evaluate the contribution of each core component in the proposed SDAM-Net architecture to the N and S prediction tasks. The structural evolution progressed through five continuous stages from a basic single-task baseline model to the final SDAM-Net. Stage 1 utilized a baseline single-task network configuration with MCNN alone for single-element prediction to establish a reference benchmark. Stage 2 adopted a multi-task network framework with a unified structure for the concurrent regression prediction of N and S *via* two regression heads. Stage 3 integrated a DCA module before the regression heads. Stage 4 introduced a shared serial global attention (S-GA) mechanism with the MCNN. Stage 5 presented the complete SDAM-Net framework. The final architecture incorporated P-GA with the MCNN and added MCA to replace the shared global module. The root mean square error (RMSE) on the test set served as the main quantitative evaluation metric.

### 2.7 Decoupling strategy interpretation

The research introduced the SHAP interpretability module.^64^ This module quantified the contribution of individual spectral features to the dual-element prediction task. The SHAP algorithm originated from cooperative game theory. The assessment of model outputs across all possible feature combinations determined the marginal contribution of each feature. The normalized fluorescence spectral sequences within the 251 to 400 nm wavelength range constituted the input features. The SHAP module calculated specific attribution scores for each wavelength point in the parallel regression tasks of N and S. A mapping process returned the extracted SHAP values to the original spectral response curves.^52^ This implementation process transformed the high-dimensional feature weights from the SDAM-Net learning phase into a one-dimensional spectral allocation. This transformation provided a quantitative validation basis. The validation basis correlated the mathematical decoupling process with the corresponding photophysical response bands.

## 3. Results and discussion

### 3.1 Physics-informed CWGAN

**Fig. 1:**
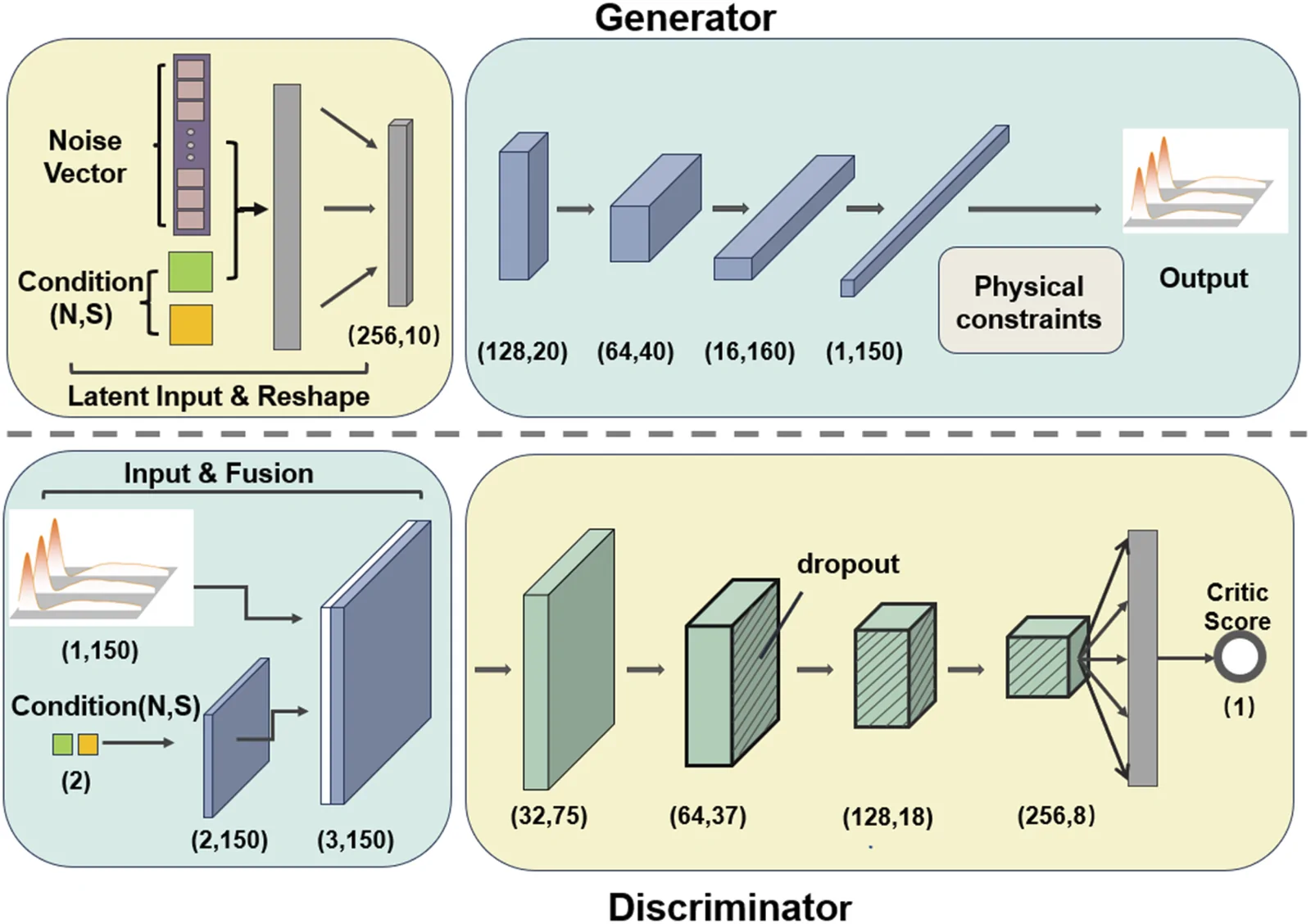
Schematic of the physics-informed CWGAN architecture.

Fig. 1 presents the spectral data augmentation network architecture based on the physics-informed CWGAN, and this network simulates authentic spectral images under physical limitations, introduces N and S conditional variables to match correct N–S combination values with the corresponding genuine spectral images, and generates simulated spectral data for arbitrary N–S combinations within the defined range to supplement missing combination cases in the original dataset. Large-kernel convolutional networks construct the generator network and the discriminator network, and the target N and S element concentrations function as conditional labels at the input terminals of both networks. The generator receives a combined vector of random noise and concentration condition labels to map and produce simulated fluorescence spectral data, and the discriminator assesses the authenticity degree of the input spectrum and determines the exact coupling match between spectral features and concentration condition labels. A gradient penalty term is incorporated into the loss function of the discriminator to enforce the Lipschitz continuity condition and guarantee the stability of the entire training process of the adversarial network. The conditional generation strategy alleviates combination sparsity regarding N and S concentrations in the experimental dataset, and the prediction instability caused by sparse sample regions is eliminated by this strategy. The physical restrictions originate from the peak response quenching laws caused by variations in the N and S content of marine fuel oil in the fluorescence spectra of tin dioxide quantum dots, and the integration of authentic physical constraints ensures strict consistency between the generated spectral data and fundamental physical laws. The physics-informed CWGAN spectral data augmentation network expands the scarce sample size and retains the characteristic fluorescence quenching laws of SnO_2_ QDs under diverse element concentration interactions.

**Fig. 2:**
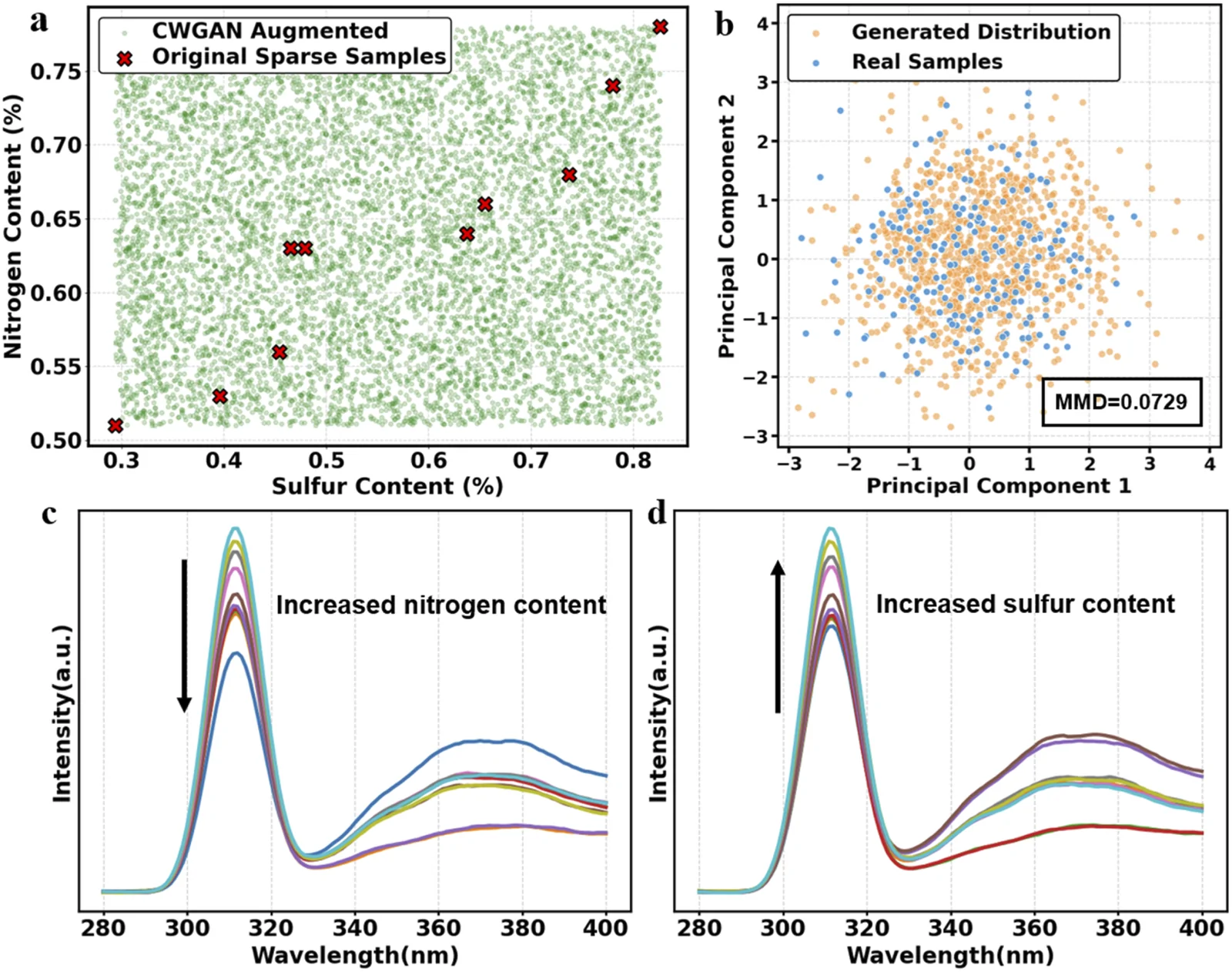
(a) N and S two-dimensional concentration space map; (b) PCA distribution diagram of the generated data and the real data; (c) S-content gradient-generated spectra; and (d) N-content gradient-generated spectra.

Fig. 2 shows the excellent performance of the CWGAN model in generating simulated spectral data. Fig. 2(a) reveals that the original data sample points encompass limited and sparse N and S combinations. The data points augmented by the CWGAN model achieve uniform and dense filling of N and S content combinations across the entire prediction interval. This filling process alleviates the unstable prediction performance of deep learning networks caused by small sample data. Fig. 2(b) demonstrates that the feature distribution of the simulated spectra overlaps spatially with that of the real samples in the two-dimensional coordinate system formed by the first principal component (PC1) and the second principal component (PC2). To quantitatively evaluate the coupling degree of these data manifolds, the maximum mean discrepancy (MMD) was calculated for the distributions in the PCA feature space. The calculated MMD score is 0.0729. This low figure demonstrates a strong coupling relationship between the principal components of the generated dataset and the real dataset. The coupling of these PCA distributions proves that the conditional generative model is capable of interpreting the core features of high-dimensional spectral data and reconstructing the spatial manifold of authentic fluorescence spectra. Fig. 2(c) and (d) show that the peak values of the generated spectra decrease with an increase in the S content gradient and increase with a decrease in the N content gradient under the condition of a constant content for the other elements. The generated spectra conform to the physical principles of the real spectra by incorporating physical constraints into the network architecture. The augmented high-quality dataset guarantees generalization ability and robustness of the subsequent task network during concentration decoupling and parallel prediction processes of the target elements.

### 3.2 SDAM-net

**Fig. 3:**
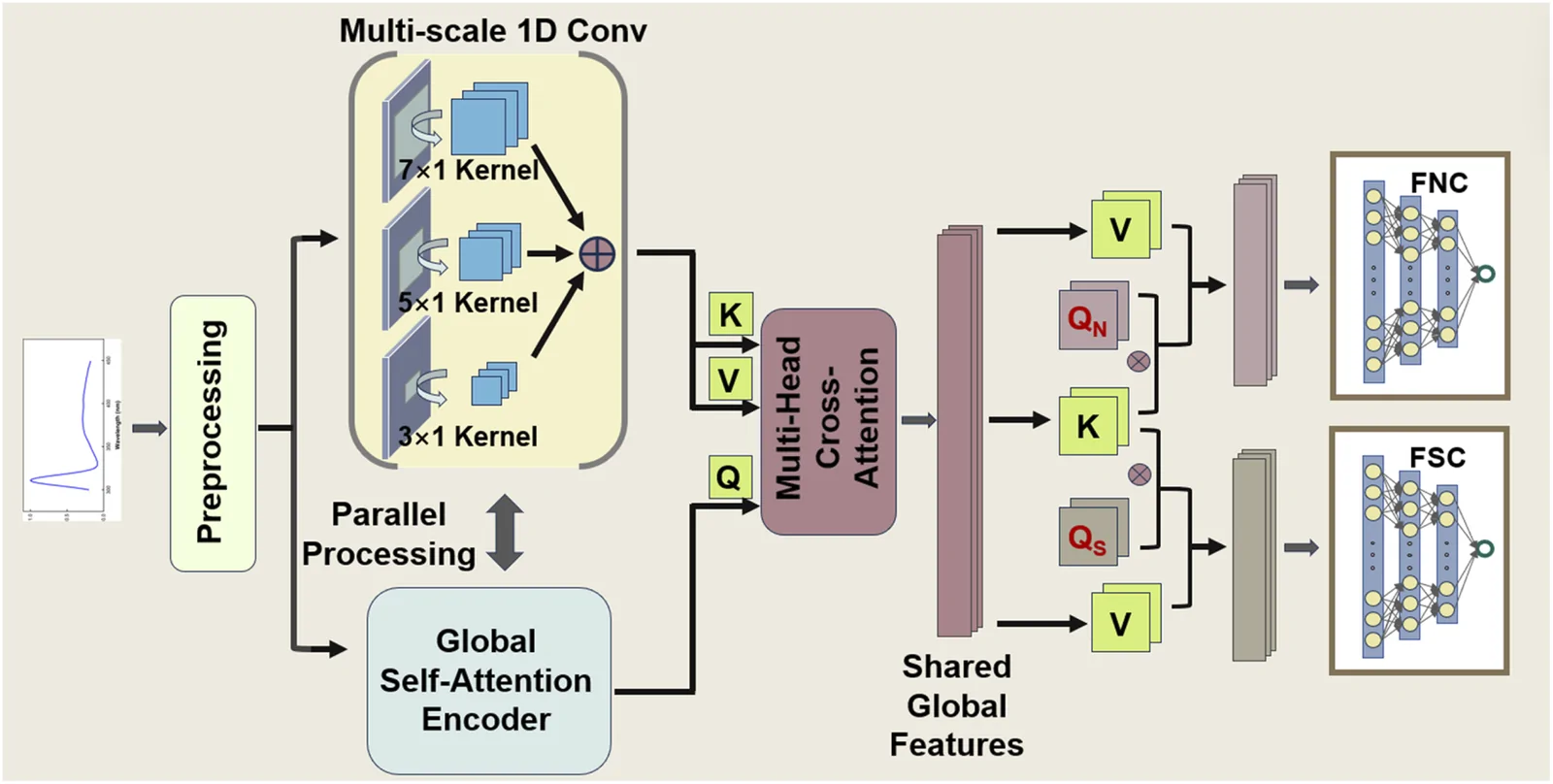
Composition of the core SDAM-Net backbone.

Fig. 3 demonstrates the integration of an attention mechanism for multi-scale feature fusion in SDAM-net. This fusion, coupled with multi-task processing, enables the decoupling of N and S signal responses. Specifically, the input spectral data undergo normalization preprocessing and are fed into the MCNN and P-GA head within SDAM-net. The branch structure of the parallel MCNN achieves local and global feature extraction across diverse receptive fields to capture microscopic fluorescence peak details and macroscopic background baseline variations. The global attention head captures the long-range dependence of the full-band spectrum to identify prominent core luminescence regions. The output features of both components flow into MCA. The global attention head performs adaptive weight allocation on the multi-scale features extracted by the MCNN within the MCA module to emphasize notable chemical response signals. The allocated and fused high-dimensional feature stream are fed into the DCA head. The DCA head executes independent attention resource allocation for the N and S regression tasks and generates differentiated feature weight matrices for both targets. The coupled N and S spectral response signals are decomposed into two non-overlapping feature subspaces, and the elemental cross-interference within the complex matrix is decoupled under this structural isolation. The independent feature data carrying single-element feature weights are fed into the regression heads of the corresponding elements. The parallel regression heads execute the final non-linear mappings to translate the high-dimensional spectral feature representations into continuous concentration values. This SDAM-Net processing yields parallel and precise quantitative prediction results for N and S concentrations in marine fuel oil.

**Fig. 4:**
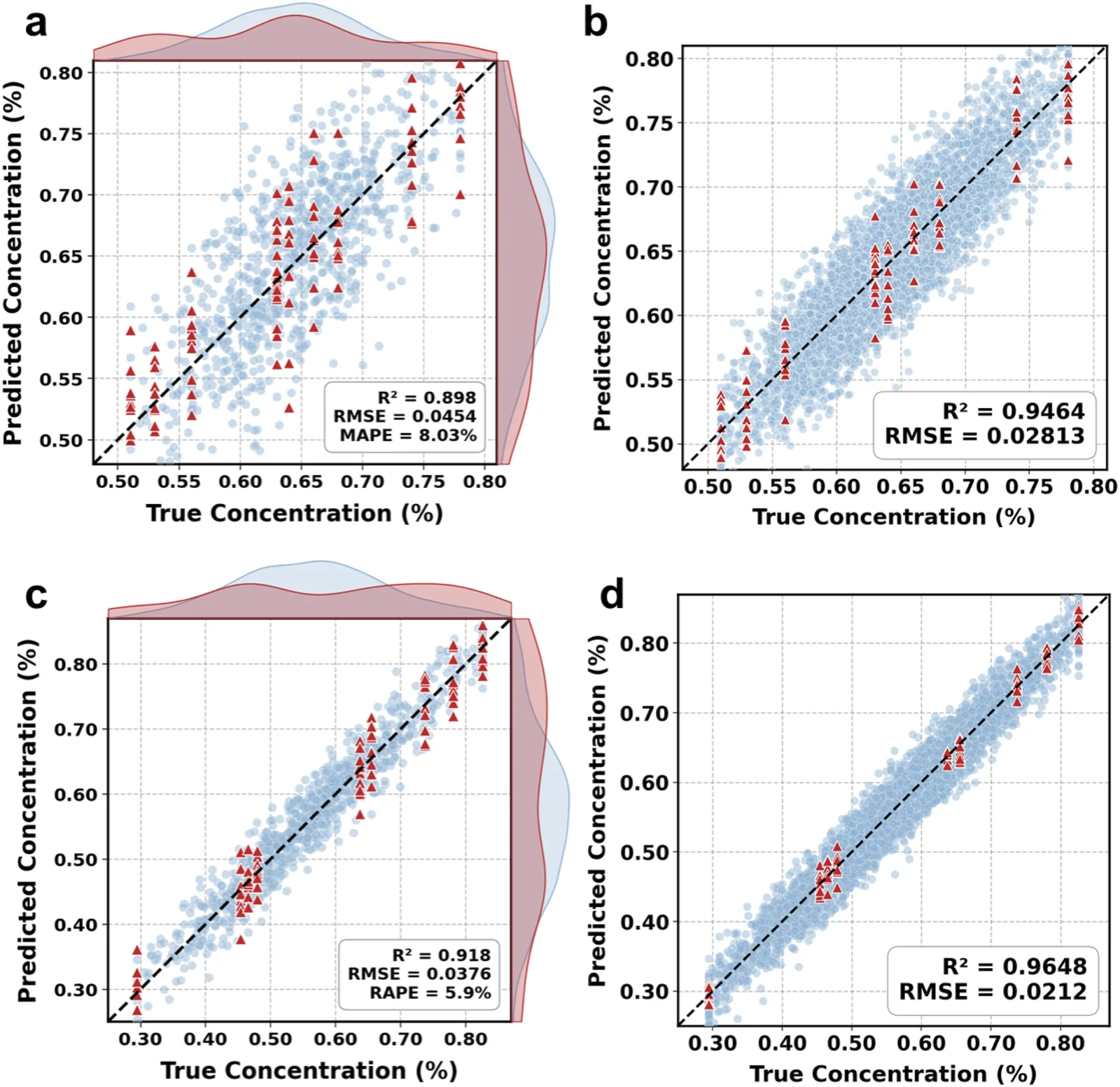
(a) Regression scatter plots of N in the test set; (b) regression scatter plots of N in the training set; (c) regression scatter plots of S in the test set; and (d) regression scatter plots of S in the training set.

Fig. 4 demonstrates the strong concurrent quantitative capability of SDAM-Net in processing fluorescence spectroscopy data with coupled N and S response signals. The predictive coefficients of determination (*R*^2^) of the model are 0.92 for S and 0.89 for N on the test set. The RMSE metrics drop to 0.0376 for S and 0.0454 for N. The mean absolute percentage error (MAPE) values remain below 8.0%, with specific metrics of 5.9% for S and 8.0% for N. The predictive performance difference between the training set and the test set stays within an appropriate range. This consistency indicates the absence of overfitting and non-convergence issues in the model.

The quantitative performance of the SDAM-Net framework was evaluated by determining its limit of detection (LOD) and limit of quantification (LOQ). Global error metrics within deep learning regression architectures exhibit a tendency to overestimate baseline noise due to the heteroscedasticity of prediction errors at elevated concentration ranges. Approximating the background noise factor using the local absolute prediction error at the lowest calibration concentration establishes a more rigorous evaluation baseline. Integrating the minimal actual fuel sample concentrations of 0.294% for S and 0.51% for N with the mean absolute error (MAE) of the model determines the prediction variance in the low-concentration region. Calculations based on standard statistical criteria of 3.3 times and 10 times the background noise yielded theoretical LODs of 0.057% for S and 0.135% for N, alongside corresponding LOQs of 0.174% for S and 0.408% for N. Both minimum concentration boundaries of the actual samples, 0.294% and 0.51%, remain above these theoretical quantification limits. These computational results confirm that the proposed decoupled network architecture possesses the requisite sensitivity for *in situ* trace detection within complex marine fuel matrices.

### 3.3 Network performance improvement

**Fig. 5:**
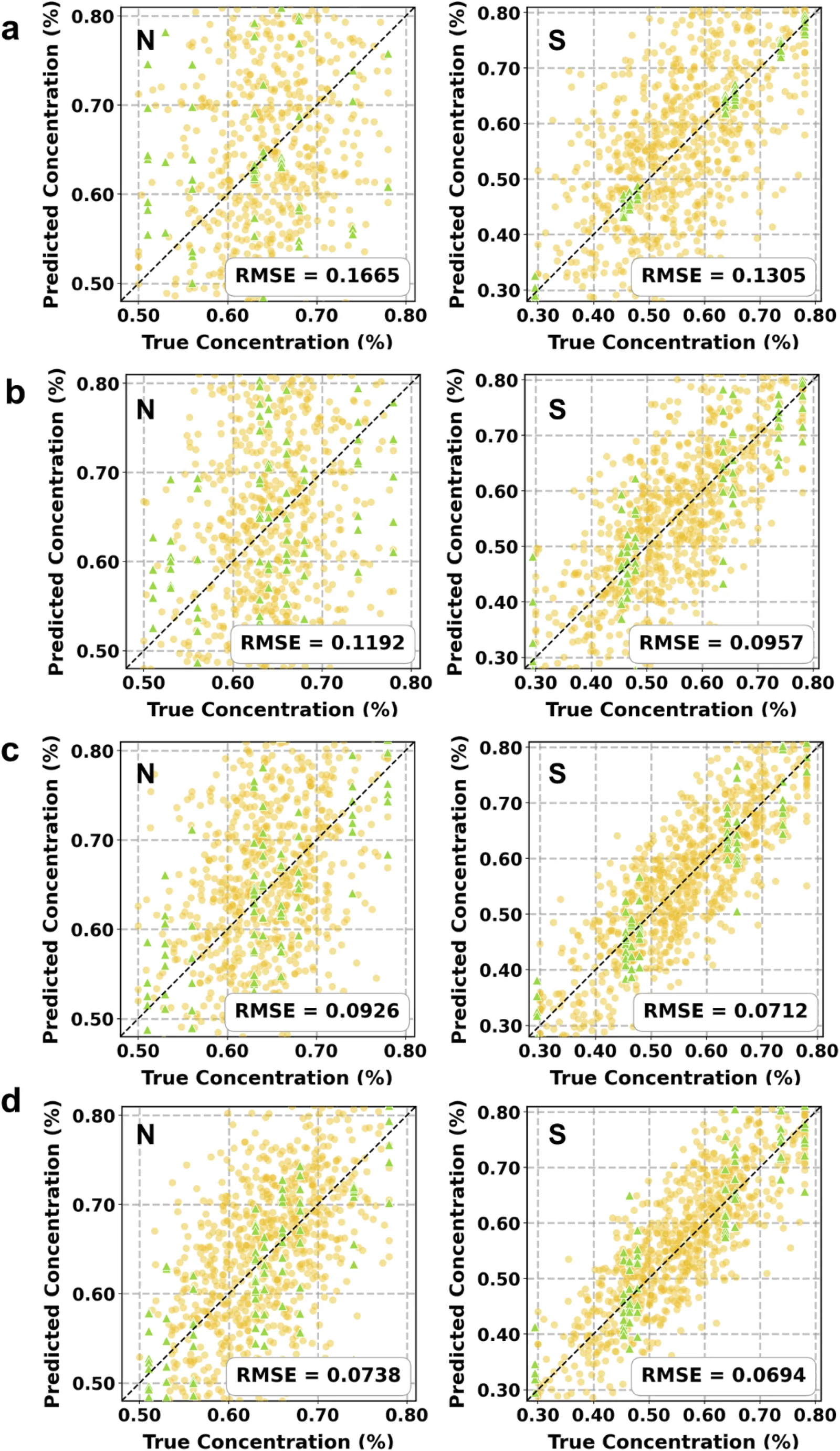
Regression scatter plots of N and S under (a) single-task baseline with the MCNN; (b) multi-task framework with the MCNN; (c) multi-task framework integrating the MCNN followed by the DCA module; and (d) multi-task framework combining the MCNN and S-GA followed by the DCA module.

Fig. 5 and Table 1 demonstrate the quantitative performance metrics across five distinct network configurations to verify the individual contributions of the core modules within the proposed SDAM-Net framework. Fig. 5(a) indicates that the baseline MCNN single-task network demonstrates limited predictive capability for the two target elements. The baseline RMSE value of S is 0.1035, and the RMSE value of N is 0.1192. Fig. 5(b) demonstrates that the shared feature extraction layer of the multi-task framework has a negative transfer effect. This phenomenon causes asymmetric performance in the two prediction tasks. The prediction of S gains a minor improvement, whereas the prediction accuracy of N suffers a substantial decrease. The dominant S signal dictates the update of shared parameters during the synchronous training process and suppresses the extraction of high-contribution feature parameters for the N prediction task. Fig. 5(c) demonstrates that the DCA module resolves the negative transfer problem. This module activates dynamic feature selection for specific task heads. The RMSE value of S reaches 0.0694, and the RMSE value of N recovers to 0.0926. Fig. 5(d) demonstrates that the shared S-GA mechanism establishes comprehensive contextual correlations. This mechanism captures long-range spectral dependencies across the entire wavelength range to achieve feature extraction. The S-GA module provides minimal improvement to the network performance. The preceding MCNN filters and discards redundant or irrelevant spectral features during the convolution process to compress local representations. This extensive feature filtering behavior causes a severe loss of global spectral context information. The subsequent serial self-attention layer fails to perform effective global self-attention encoding due to a lack of sufficient original feature information. The uniform weight allocation on the remaining compressed bands triggers a certain degree of feature dilution.

**Table 1:** Performance comparison of various network structures

Model	Index	Testing set			
		*R* ^2^	RMSE	MAE	MAPE
Single-task + MCNN	S	0.79	0.1035	0.0828	17.3%
	N	0.74	0.1192	0.1332	18.6%
Multi-task + MCNN	S	0.82	0.0957	0.0766	16.5%
	N	0.63	0.1665	0.0954	27.2%
Multi-task + MCNN + DCA	S	0.86	0.0712	0.0573	11.7%
	N	0.79	0.0926	0.0741	15.1%
Multi-task + MCNN + S-GA + DCA	S	0.86	0.0694	0.0557	9.4%
	N	0.83	0.0738	0.0590	11.9%
**Multi-task + MCNN + P-GA + MCA + DCA**	**S**	**0.92**	**0.0376**	**0.0301**	**5.9%**
	**N**	**0.89**	**0.0454**	**0.0363**	**8.0%**

Table 1 demonstrates that the complete SDAM-Net configuration achieves the optimal performance among all the evaluated variants. The P-GA and MCA modules replace the serial shared structure to maximize the feature extraction capability. The P-GA path extracts long-range dependencies of specific targets from the pre-convolution spectral data. This extraction avoids information loss from the MCNN and preserves diverse feature characteristics before the multi-scale spatial downsampling process. The MCA module regulates the dynamic feature interaction between the parallel path and the convolution stream to achieve optimal information fusion. The final architecture generates a maximum *R*^2^ of 0.92 for S and 0.89 for N and the minimum RMSE values reach 0.0376 for S and 0.0454 for N.

### 3.4 Spectral feature assignment

**Fig. 6:**
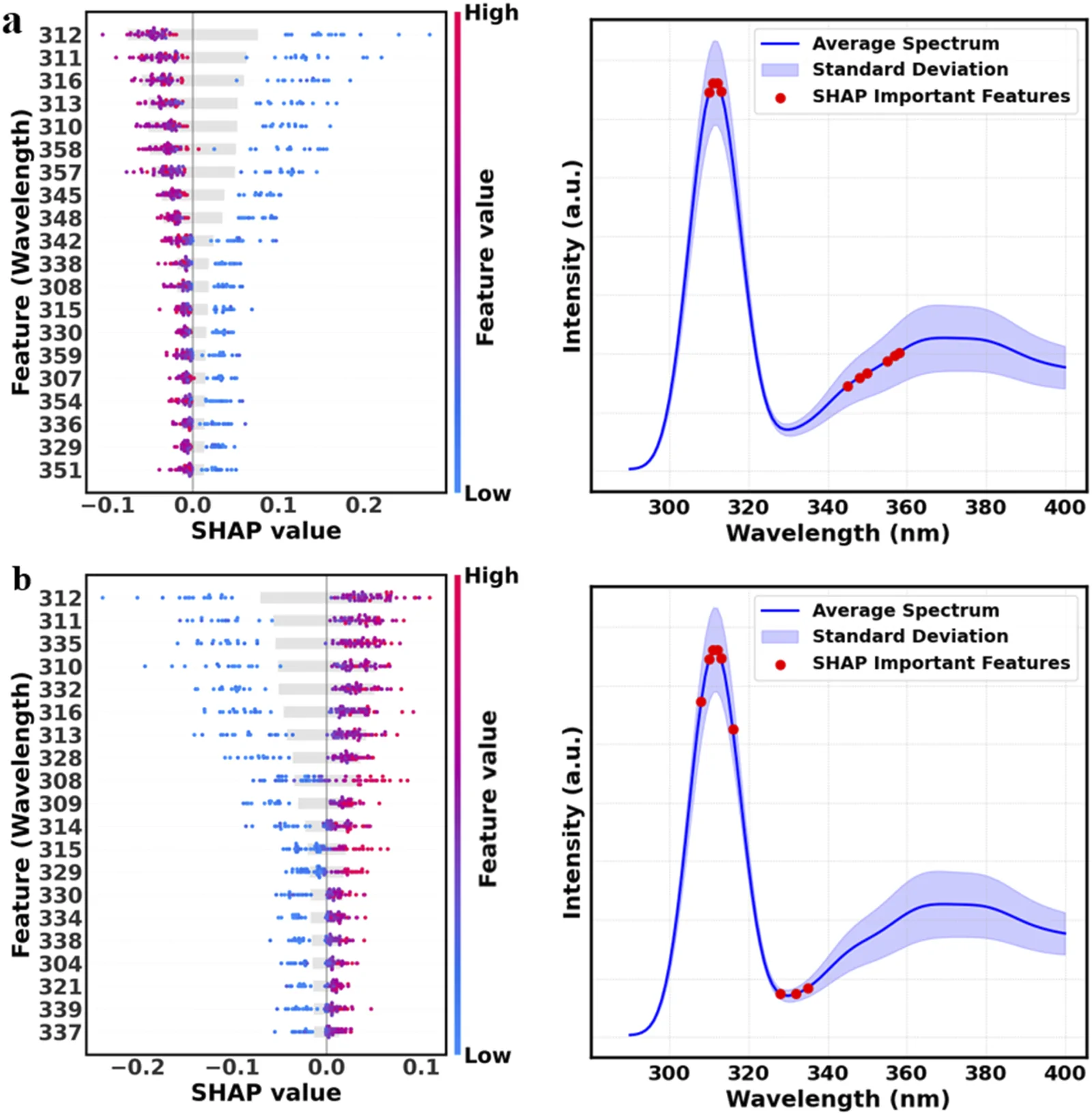
Significant features of (a) N and (b) S prediction mapped to real spectral data.

Fig. 6 displays the accurate assignment of significant features from the N and S prediction tasks to the real fluorescence response spectra through the SHAP module. This assignment restores the interpretability of the network decoupling process at the physical level.

Fig. 6(b) reveals that the network focuses exclusively on the main fluorescence emission peak in the S detection task. This concentrated attention stems from the profound and irreversible fluorescence quenching induced by S-containing species.^19^ S acts as a typical soft base and exhibits a strong chemical affinity for the hard acid Sn^4+^ on the surface of SnO_2_ QDs.^65^ This interaction surpasses simple physical adsorption. The process causes severe chemical destruction of the surface lattice and initiates the generation of a non-radiative SnS_*x*_ dead layer. The strong electron-donating capability of S injects a massive amount of electrons into the conduction band. This injection triggers an intense Auger recombination.^66^ The model assigns a negligible weight to the subsequent long-wavelength region through an adaptive mechanism. Small molecule sulfides act as pure electron donors in the standard photoinduced electron transfer (PET) process. These molecules produce no substantial perturbation in the local crystal field or dielectric environment. The long-wavelength spectrum undergoes proportional amplitude scaling in tandem with the attenuation of the main peak. This scaling provides no new information entropy. The network identifies multicollinearity.

Fig. 6(a) demonstrates the allocation of significant weights to the main emission peak and the long-wavelength broad peak in the 350 to 400 nm range during the N prediction task. N-containing functional groups initiate PET-based dynamic quenching in the main peak region. An abnormal fluorescence recovery phenomenon appears in the higher concentration range from 0.5% to 0.7%.^20^ N atoms act as hard bases to achieve deep surface passivation of the quantum dots. This passivation forms an effective coordination with Sn^4+^ sites. The coordination eliminates surface dangling bonds and passivates non-radiative electron traps. The dense surface coordination of macromolecular N-containing heterocycles, including quinoline, acridine, and carbazole derivatives,^67^ coupled with bulky aliphatic amines, induces substantial steric hindrance. This spatial obstruction establishes a robust physical barrier around the nanocrystals to isolate adjacent quantum dots and suppress the aggregation-caused quenching (ACQ) effect. A rigid solvation shell emerges to suppress non-radiative thermal energy dissipation. The high reliance of the network on the long-wavelength spectral segment in N prediction reveals complex surface topological deformations. The Lewis base strength of nitrogenous species depends on the availability of their lone electron pairs. N moieties possessing localized lone pairs, encompassing pyridinic and aliphatic amine groups, exhibit intense Lewis basicity.^68^ This potent basicity drives the formation of robust chemical coordination with surface metal active sites. This process induces local geometric distortion of the crystal lattice. This deformation alters the original crystal field splitting. The alteration activates deep oxygen vacancy (V_o_) traps. The intrinsic low-energy radiative emission of these defects manifests as a broad peak in the 350 to 400 nm range.^69^ The adsorption layer constructed by macromolecular N-containing polycyclic aromatic hydrocarbons causes a sudden change in the interface dielectric constant. This layer forms a local microelectric field. This altered environment reduces the exciton Coulomb binding energy through the quantum-confined Stark effect.^70^ This reduction translates into a Stark redshift and inhomogeneous broadening in the macroscopic emission spectrum.

The SHAP feature assignment validates the precise distinction between the symmetrical scaling features of S and the local Stark broadening and defect activation features of N. This assignment confirms the strict alignment of the SDAM-Net attention mechanism with the distinct photophysical quenching mechanisms of the two elements. The network captures and decouples the dominant spectral variation feature parameters.

### 3.5 Generalization ability and robustness

The limited volume of authentic spectral samples poses a structural overfitting risk. The physics-informed CWGAN solves this fundamental data sparsity through the reconstruction of a continuous spectral manifold. This generative approach anchors the predictive model to photophysical principles and prevents memorization of empirical noise. Internal network regularization prevents overfitting and improves computational robustness. A 20% dropout mechanism within the multi-layer perceptron and an L2 penalty *via* the AdamW optimizer restrict excessive parameter adaptation. A ten-fold cross-validation protocol confirms the structural stability and predictive accuracy of SDAM-Net within the boundaries of the established dataset. However, existing laboratory conditions constrain current evaluations. The reliance on a single batch of marine fuel oil and a fixed fluorescence spectrophotometer restricts the diversity of matrix backgrounds and instrumental noise. For this reason, the general applicability of the network model under distinct environmental conditions or alternative hardware system warrants further improvement.

The integration of explicit feature engineering facilitates the expansion of generalization boundaries. Targeted feature extraction methodologies, incorporating principal component analysis, peak fitting, and autoencoders, provide robust solutions for data representation.^71^ Although the proposed SDAM-Net operates as an end-to-end deep learning model, a hybrid input strategy based on engineered features captures more robust data manifolds. The feature weights derived from the SHAP interpretability module guide the identification of critical spectral zones. The manual extraction of specific chemical descriptors, such as peak intensities, peak shifts, full widths at half maximum, or spectral derivatives, and the subsequent combination of these engineered parameters with raw spectral intensities into a multidimensional dataset for network training can yield a stronger representation. This multidimensional combination minimizes the risk of overfitting to specific instrumental configurations and advances the generalization adaptability of the detection platform.

## 4. Conclusion

The SDAM-Net architecture utilizes attention mechanisms to achieve multi-scale feature fusion. This network integrates multi-task mechanisms to accomplish the decoupling of the response signals of N and S in the fluorescence spectra of SnO_2_ QDs. The incorporation of a “Multi-task + MCNN + P-GA + MCA + DCA” structure resolves the negative transfer problem between the two prediction tasks. The framework realizes the efficient *in situ* synchronous detection of N and S elements in marine fuel oil. The test set evaluation results confirm the superiority of this network architecture in terms of detection accuracy within the specific concentration ranges of 0.51% to 0.78% m/m for N and 0.294% to 0.826% m/m for S. The quantitative prediction RMSEs for N and S reach minimum values of 0.0454% and 0.0376%, respectively. These metrics demonstrate prediction precision beyond standard network models. The physics-informed CWGAN model provides vital auxiliary support to accomplish precise reconstruction of the continuous spectral manifold. The conditional generation strategy alleviates the sparsity of the combination concentration. This process executes the elimination of the prediction instability caused by the sample combination of scarce regions. The introduction of the SHAP explanation module assigns prominent features to the authentic microscopic spectral responses. This assignment process offers intuitive confirmation of the successful decoupling of entangled spectra. The validation confirms that the model's decoupling strategy aligns with the underlying physics. The SDAM-Net framework overcomes the bottleneck of existing *in situ* technologies regarding synchronous dual-element detection. This framework presents extensive applicability in complex coupled signal processing. This method establishes a robust new paradigm for the *in situ* efficient quantitative detection of N and S in marine fuel oil and provides a crucial detection tool for global atmospheric environmental protection and governance.

## Author contributions

Haoze Tian: writing – original draft, investigation, conceptualization. Yanan Zhang: methodology, investigation. Di Wu: methodology, investigation. Chuqiao Hu: data curation. Peilun Qiu: Writing – review and editing. Jianqiao Liu: Writing – review and editing, supervision. Ce Fu: writing – review and editing, funding acquisition.

## Conflicts of interest

The authors declare that they have no known competing financial interests or personal relationships that could have appeared to influence the work reported in this paper.

## Data Availability

Data will be made available on request.
